# Interleukin 6 Present in Inflammatory Ascites from Advanced Epithelial Ovarian Cancer Patients Promotes Tumor Necrosis Factor Receptor 2-Expressing Regulatory T Cells

**DOI:** 10.3389/fimmu.2017.01482

**Published:** 2017-11-06

**Authors:** Nirmala Chandralega Kampan, Mutsa Tatenda Madondo, Orla M. McNally, Andrew N. Stephens, Michael A. Quinn, Magdalena Plebanski

**Affiliations:** ^1^Department of Immunology and Pathology, Monash University, Melbourne, VIC, Australia; ^2^Oncology Unit, Royal Women’s Hospital, Melbourne, VIC, Australia; ^3^Department of Obstetrics and Gynaecology, Pusat Perubatan Universiti Kebangsaan Malaysia, Kuala Lumpur, Malaysia; ^4^Centre for Cancer Research, Hudson Institute of Medical Research, Clayton, VIC, Australia; ^5^Department of Molecular and Translational Sciences, Monash University, Clayton, VIC, Australia; ^6^Epworth Research Institute, Epworth Healthcare, Richmond, VIC, Australia; ^7^School of Health and Biomedical Sciences, RMIT University, Melbourne, VIC, Australia

**Keywords:** epithelial ovarian cancer, malignant ascites, interleukin 6, tumour necrosis factor 2, FoxP3, regulatory T cells, effector T cells, inflammation

## Abstract

**Background:**

Epithelial ovarian cancer (EOC) remains a highly lethal gynecological malignancy. Ascites, an accumulation of peritoneal fluid present in one-third of patients at presentation, is linked to poor prognosis. High levels of regulatory T cells (Tregs) in ascites are correlated with tumor progression and reduced survival. Malignant ascites harbors high levels of Tregs expressing the tumor necrosis factor receptor 2 (TNFR2), as well as pro-inflammatory factors such as interleukin 6 (IL-6) and tumor necrosis factor (TNF). IL-6 is also associated with poor prognosis. Herein, we study the effect of IL-6 and TNF present in ascites on the modulation of TNFR2 expression on T cells, and specifically Tregs.

**Methods:**

Ascites and respective peripheral blood sera were collected from 18 patients with advanced EOC and soluble biomarkers, including IL-6, sTNFR2, IL-10, TGF-β, and TNF, were quantified using multiplexed bead-based immunoassay. Peripheral blood mononuclear cells (PBMC) from healthy donors were incubated with cell-free ascites for 48 h (or media as a negative control). In some experiments, IL-6 or TNF within the ascites were neutralized by using monoclonal antibodies. The phenotype of TNFR2^+^ Tregs and TNFR2^−^ Tregs were characterized post incubation in ascites. In some experiments, cell sorted Tregs were utilized instead of PBMC.

**Results:**

High levels of immunosuppressive (sTNFR2, IL-10, and TGF-β) and pro-inflammatory cytokines (IL-6 and TNF) were present in malignant ascites. TNFR2 expression on all T cell subsets was higher in post culture in ascites and highest on CD4^+^CD25^hi^FoxP3^+^ Tregs, resulting in an increased TNFR2^+^ Treg/effector T cell ratio. Furthermore, TNFR2^+^ Tregs conditioned in ascites expressed higher levels of the functional immunosuppressive molecules programmed cell death ligand-1, CTLA-4, and GARP. Functionally, TNFR2^+^ Treg frequency was inversely correlated with interferon-gamma (IFN-γ) production by effector T cells, and was uniquely able to suppress TNFR2^+^ T effectors. Blockade of IL-6, but not TNF, within ascites decreased TNFR2^+^ Treg frequency. Results indicating malignant ascites promotes TNFR2 expression, and increased suppressive Treg activity using PBMC were confirmed using purified Treg subsets.

**Conclusion:**

IL-6 present in malignant ovarian cancer ascites promotes increased TNFR2 expression and frequency of highly suppressive Tregs.

## Introduction

Ovarian cancer is one of the most lethal types of cancer in women globally (1, 2). This is because the majority of ovarian cancer patients are diagnosed in late stages, with up to one-third of patients presenting with a prominent peritoneal accumulation of fluid called “ascites.” Ascites development is associated with chemo-resistance, disease recurrence (3, 4), and poorer survival in ovarian cancer patients (5–9). Ovarian cancer ascites further contains a complex reservoir of immune cells and cytokines, harboring immunosuppressive cells as well as inflammatory soluble factors (7, 10, 11). This unique milleau has been proposed to help tumor cells evade host immunosurveillance, so that tumor cells can continue growing without restriction (3, 7, 9, 10). In ovarian cancer, similar to other cancers, the immune system is hampered in controlling the tumor due to the presence of regulatory T cells (Tregs) that inhibit T effector (Teff) cell-mediated antitumor responses (9).

Tumor necrosis factor receptor type II (TNFR2) stimulates the activation and proliferation of Tregs from a resting to an activated state (12). Expression of TNFR2 on Tregs is reported to identify the maximally suppressive and functional Treg population in both mice and humans (13–15). Overabundance of TNFR2^+^ Tregs creates a potent immunosuppressive microenvironment associated with negative patient outcomes in diverse cancers, such as acute myeloid leukemia, lung cancer, ovarian cancer, and colorectal cancer (16–20). Decreasing TNFR2^+^ Treg levels using cyclophosphamide in mice (21) or panobinostat and azacitidine in humans (19) is associated with improved antitumor immune responses and prolonged survival. Lenalidomide has also been shown to both decrease TNFR2^+^ Treg levels and enhance Teff function in patients with acute myeloid leukemia. The high levels of TNFR2^+^ Tregs in ovarian cancer ascites can be driven by their preferential migration into the ascites, given their high levels of expression of the CCR4 chemokine receptor (18). It is also possible that cytokines present in ascites may promote TNFR2 expression on Tregs. Once TNFR2 is expressed on Tregs, tumor necrosis factor (TNF) in ascites can further stabilize FoxP3 expression, the hallmark transcription factor associated with Treg suppressive capabilities (22).

Tumor necrosis factor receptor 2 expression is elevated on peripheral blood mononuclear cells (PBMCs) of ovarian cancer patients, as well as on mononuclear cells present in ovarian cancer ascites (18). In a previous study looking at TNFR2^+^ Tregs from ovarian cancer ascites, Govindaraj and colleagues observed that TNFR2^+^ Tregs extracted from ascites express higher levels of immunosuppressive molecules CTLA-4 and GARP, and are functionally more suppressive when compared to peripheral blood TNFR2^+^ Tregs (18). Induction of CTLA-4 and GARP expression on human Tregs is dependent on the transcription factor FoxP3 (23–26). In the present study, we have assessed whether soluble components present in cell-free ascites can promote upregulation of these functional immunosuppressive molecules on Tregs, and their association with a TNFR2^+^ Treg phenotype.

Apart from these immunosuppressive check-point inhibitor receptors, it is also important to assess whether ascites may modulate the expression of other immunosuppressive molecules currently being explored for ovarian cancer immunotherapy. Programmed cell death ligand-1 (PD-L1), a member of B7 superfamily, is a negative immunoregulatory molecule that inhibits effector T cell activity and is highly expressed on cancer cells (27–31) and immune cells including Tregs (32, 33). PD-L1 expression on Tregs is associated with upregulated FoxP3 expression and promotes maximally suppressive Treg activity (34). PD-L1 inhibitors have demonstrated promising antitumor efficacy in several cancer types, including melanoma, non-small cell lung cancer, renal cell carcinoma, bladder carcinoma, and Hodgkin’s lymphoma (35–37). Recent studies have shown that PD-L1 can be upregulated by pro-inflammatory cyokines such as TNF (38).

Elevated interleukin 6 (IL-6) in ascites and in the serum of patients with advanced ovarian cancer has been most strongly correlated with poor survival (39–41) as it has in multiple other cancers (42). IL-6 is a pleiotropic cytokine and an essential biomarker in the cytokine cascade that is involved in the initiation and regulation of inflammation (43, 44). It can be synthesized by dendritic cells, macrophages (45, 46), lymphocytes (47–50), somatic cells (e.g., fibroblasts, keratinocytes, endothelial cells) (51–53), and multiple cancer cell types including breast, lung, head and neck, colorectal, hepatobiliary, pancreatic, as well as ovarian cancer cells (54–62).

Accumulated Tregs in ascites and tissue have been found to be higher in patients with ovarian cancer and linked to advanced ovarian disease and poor prognosis (9). The production of TNF and IL-6 are concomitantly increased in these conditions (63); therefore, the potential effect of these pro-inflammatory cytokines on Tregs is of interest. The relationship between Tregs, IL-6, and TNF is likely to be complex. Experiments using murine cells with autoimmune disease reported that TNF promotes proliferation and maintains suppressive activity of Treg cells both *in vitro* and *in vivo* (13, 64). In contrast, there are conflicting reports of the activity of TNF on human Tregs. Some studies suggest that TNF promotes a reduction in the expression of FoxP3 and inhibits the suppressive activity of human Tregs (65, 66). Conversely, a recent study showed that TNF, in the presence of IL-2, increases the expression of human Tregs (both CD25 and FoxP3), and their suppressive activity in a 3-day culture (67). TNFR2 is agreed to be the primary receptor for TNF on both murine and human Treg cells.

The effect of IL-6 on Tregs similarly has been a source of significant controversy. IL-6 has been reported to promote differentiation into T helper type 2 differentiation cells (68) and influence the balance between IL-17 producing cells (Th17) and Tregs (69). While IL-6 alone is unable to induce Th17 cells, culturing of IL-6 in combination with TGF-β (70–73) has been reported to promote murine and human naïve T cells to become Th17 and inhibit conversion into Tregs. In contrast, inducible Tregs activated in the presence of IL-2 and TGF-β did not differentiate into Th17 when cultured with IL-6 (74). In a murine study mimicking excessive IL-6 as seen in chronic inflammatory disorders and several cancers, T cells isolated from peripheral lymphoid organs in IL-6 transgenic mice not only had increased levels of Th17 but also Tregs which further were shown to have retained suppressive activity (75). This *in vivo* study, therefore, suggests that excessive IL-6 conditions do not negatively affect development and function of Tregs and may potentially promote them under specific conditions (75).

To explore the relationship between Tregs, TNF, and IL-6 in ovarian cancer ascites, we created an *in vitro* system to study the effect of IL-6 and TNF within cell-free ovarian cancer ascites on TNFR2^+^ Treg and on TNFR2^+^ Teff frequency and function. Our results suggest a critical role for IL-6, present in ovarian cancer ascites, in promoting highly functional TNFR2^+^ Tregs, which are shown to be the only Treg subset capable of suppressing TNFR2^+^ Teffs in ovarian cancer ascites cultures.

## Materials and Methods

### Trial Design and Patient Details

This study was carried out in accordance with the recommendations of an Immunity and Ovarian Cancer trial (Project 13/32), HREC of Royal Women’s Hospital with written informed consent from all patients. All patients gave written informed consent in accordance with the Declaration of Helsinki. The protocol was approved by the HREC of the Royal Women’s Hospital, Melbourne. Ascites and peripheral blood serum samples were prospectively obtained from 18 patients with newly diagnosed advanced epithelial ovarian cancer (EOC) seen in the Oncology Unit, Royal Women’s Hospital, Melbourne, Australia following informed consent. All relevant clinical information including demographic status, medical and drug history, clinical diagnosis, and disease extent and status were prospectively collected. Blood samples were obtained immediately prior to surgery and general anesthesia. Ascites samples were collected either during peritoneal tapping prior to chemotherapy or at the time of surgery. Histologic diagnosis of the study patients was confirmed independently by senior hospital pathologists, and all histologic data were prospectively collected and stored in a computerized hospital database. For healthy blood samples, 40 buffy coats were obtained from blood donated by healthy adult volunteers acquired at Australian Red Cross Blood Bank Service.

### Isolation of Peripheral Blood Serum

Pre-operative venous blood was drawn from ovarian cancer patients into serum collecting (SST) vacutainer tubes (BD). Following collection, the tubes were left undisturbed at room temperature for 30 min to allow blood to clot. The clots were removed by centrifugation at 1,000 *g* for 10 min in a refrigerated centrifuge. All sera were stored at −80°C until use.

### Isolation of Ascites Supernatant

Ascites samples from ovarian cancer patients were first filtered through a 100-µm cell strainer and centrifuged to remove the cellular component. The cell-free supernatant layer of the ascites was collected and stored at −80°C until use.

### Isolation of PBMCs

Healthy donor (Australian Red Cross Blood Services) PBMCs were isolated by Ficoll (Amersham Pharmacia Biotech, Sweden) density gradient centrifugation. The isolated PBMCs were suspended in cryovials containing a freeze medium mixture of 10% DMSO (Sigma-Aldrich, USA) and 90% heat-inactivated fetal calf serum (GIBCO, Life Technologies, USA) and frozen at a speed of −1°C/min in a −80°C freezer then subsequently stored in liquid nitrogen. Prior to cell culture, each vial of frozen PBMCs was rapidly thawed in a 37°C water-bath and resuspended in complete AIM V media [AIM V (Life Technologies, USA) supplemented with 5% normal human serum (Sigma-Aldrich, USA)].

### *In Vitro* Conditioning with Ascites

Peripheral blood mononuclear cells from healthy donors were cultured in a 96-well culture plate with 150 μl/well of either complete AIM V media alone or with 50% ascites supernatant (obtained from patient ascites *via* centrifugation) at a final concentration of 2 × 10^6^ cells/ml. The cells were then incubated in a humidified incubator at 37°C with 5% CO_2_. After 48 h, the cells were harvested, antibody labeled, and further analyzed by flow cytometry.

### *In Vitro* Blockade of Cytokines within Ascites with Monoclonal Antibodies (mAbs)

Peripheral blood mononuclear cells from healthy donors were isolated by Ficoll density centrifugation and incubated *in vitro* in either complete AIM V media or cell-free ascites from an advanced EOC patient. IL-6 and TNF within the ascites was blocked with murine anti-human IL-6 monoclonal antibody (final concentration at 2.5 µg/ml, R&D, USA) and murine anti-human TNF monoclonal antibody at 500 ng/ml (R&D, USA). Mouse IgG1 immunoglobulins (isotype control) (R&D USA) were used as a negative control. Isotype control and mAbs were added into media and ascites, respectively, in a 10-ml tube, gently suspended and incubated at room temperature for 10 min. PBMCs from healthy donors were then added into a 96-well culture plate with 150 μl/well of either complete AIM V media alone or with 50% ascites supernatant (blocked IL-6 or TNF or both) at a final concentration of 2 × 10^6^ cells/ml. Following 48 h of incubation in a humidified incubator at 37°C with 5% CO_2_, cells were washed, stained, and analyzed with flow cytometry.

### Flow Cytometric Analysis

To determine the frequency and phenotype of T cell populations in PBMCs, multicolor flow cytometry was performed using the following surface antibodies: anti-CD3 PerCP (BD Pharmingen, USA) and anti-CD8 FITC (Biolegend, USA); anti-CD4 APC-Cy7 (BD Pharmingen, USA), and anti-CD25 PECF584 (BD Pharmingen, USA), anti-CD127 BV650 (Biolegend, USA), anti-TNFR2 biotinylated followed by conjugation with Streptavidin PECy7 (BD Pharmingen, USA), anti-PD-L1 PE (Biolegend, USA), anti-CTLA-4 BV605 (Biolegend, USA), and anti-GARP BV711 (BD Pharmingen, USA). Following primary staining, a fixable dead cell marker (Life Technologies, USA) was also used to distinguish between dead and live cells. Intracellular levels of FoxP3 and IFN-γ were determined following fixation and permeabilization of cells using a fixation/permeabilization buffer kit (eBioscience, USA) then staining the cells with anti-FoxP3 APC (eBioscience, USA) and anti- IFN-γ v450 antibody (eBioscience, USA). Flow cytometry data were acquired on a Becton Dickinson LSR II flow cytometer using FACS Diva software, acquiring a minimum of 100,000 events per sample. Fluorescence minus one (FMO) controls and isotype-matched immunoglobulins were used to enable accurate gating. Data were analyzed using Flow jo software (TreeStar, USA).

### Intracellular Cytokine Analysis

Peripheral blood mononuclear cells from healthy donors cultured in a 48-well plate at final concentration of 2 × 10^6^ cells/ml per well in either AIM V media alone or with 50% ascites supernatant for 48 h. Cells were washed and then stimulated for 6 h with 50 ng/ml of phorbol 12-myristate 13-acetate (PMA) and 1 µg/ml of ionomycin (Sigma-Aldrich, USA) at 37°C in a 5% CO_2_, humidified incubator. Brefeldin A (eBioscience, USA) was added for the last 5 h of incubation at a concentration of 1 µg/ml. After stimulation, the cells were washed and labeled for surface markers as above, followed by intracellular staining for FoxP3 and IFN-γ and then prepared for flow cytometric analysis. Unstimulated cells, FMO, and isotype-matched immunoglobulins were also used as controls. Flow cytometry data were acquired on a Becton Dickinson LSR II flow cytometer, and data were analyzed using Flowjo software (TreeStar, USA).

### Cell Sorting and Culture

Flow cytometric cell sorting was performed using BD Influx to isolate Teff and Tregs cells as well as Tregs TNFR2^+^ and Tregs TNFR2^−^ subsets. PBMCs from healthy donors were stained with the following surface antibodies: anti-CD3 PerCP (BD Pharmingen, USA), anti-CD4 APC-Cy7 (BD Pharmingen, USA), anti-CD127 BV650 (Biolegend, USA), and anti-CD25 PECF584 (BD Pharmingen, USA). For isolation of Tregs TNFR2^+^ and Tregs TNFR2^−^ subsets in some experiments, pre-sort PBMCS were additionally stained for biotinylated followed by conjugation with Streptavidin PECy7 (BD Pharmingen, USA). A fixable dead cell marker (Life Technologies, USA) was used to distinguish between dead and live cells. Following exclusion for doublet and dead cells, cells for sorting were initially gated on the CD3^+^CD4^+^ population. Within CD4^+^ gates, cells were then gated on CD127 and CD25 to identify and isolate CD25^hi^CD127^lo^ (Tregs) and CD25^−^CD127^+^ (Teff) population, respectively. Additionally, further gating on TNFR2 was performed to isolate Treg TNFR2^+^ and Tregs TNFR2^−^ subsets. Prior to culture, purity was assessed on post-sort Tregs and were confirmed to be 95 ± 3% FoxP3^+^ by flow cytometry. Samples containing the sorted populations were then suspended at a ratio of 10^5^ cells per 50 µl of complete AIM V media and were cultured in either complete AIM V media alone or in cell-free ascites for 48 h. The cells were then incubated in a humidified incubator at 37°C with 5% CO_2_. After 48 h, the cells were harvested, antibody labeled to assess phenotype, and then analyzed by flow cytometry.

### *In Vitro* Suppression Assay

To determine the difference in ratio at which Tregs incubated in ascites are suppressive compared Tregs incubated in media, add-back suppression assays were performed. Following *in vitro* conditioning of PBMCS in either complete AIM V media or ascites for 48 h, the cells were washed, harvested, and labeled with anti-CD3 PerCP (BD Pharmingen, USA), anti-CD4 APC-Cy7 (BD Pharmingen, USA), anti-CD127 BV650 (Biolegend, USA), and anti-CD25 PECF584 (BD Pharmingen, USA). Following primary staining, a fixable dead cell marker (Life Technologies, USA) was used to distinguish between dead and live cells. The cultured cells underwent cell sorting as described in Section “Cell Sorting and Culture.” Three populations consisting of Teff incubated in media, Tregs incubated in media, and Tregs incubated in ascites were isolated. Teff were further labeled with Carboxyfluorescein Diacetate Succinimidyl Ester (CSFE) (Molecular Probes, US) at 0.5 µM to monitor cell proliferation. The labeled effector cells were cultured alone, or at a 1:2, 1:4, or 1:8 and 1:16 ratio with autologous Tregs already conditioned in either complete AIM V media or ascites. The cells were cultured (usually in triplicates) in a 96-well plate pre-coated with anti-CD3 (1.0 µg/ml clone OKT3, Biolegend). This was followed by the addition of soluble anti-CD28 (1.0 µg/ml clone CD28.2, BD Pharmingen) for 3 days at 37°C with 5% CO_2_. Cells were harvested, stained, and further analyzed by flow cytometry.

### Multiplexed Bead-Based Immunoassay

Cell-free ascites were collected from 18 patients with advanced EOC and soluble biomarkers were quantified using BD™ Cytokine cytometric bead arrays (flex sets for IL-10, IFN-γ, IL-2, IL-6, TNF, TGF-β, and sTNFR2, BD USA), following manufacturer’s protocol. Ascites supernatants were used at 1 in 4 and 1 in 8 dilution. Ascites samples were analyzed in duplicates and results were calculated as the average of two values. Samples were then acquired by flow cytometry on a Becton Dickinson LSR II flow cytometer collecting 300 events per analyte. Flow cytometry standard data files were exported and was analyzed by FCAP Array software version 3.0.

For simultaneous measurement of multiple cytokines in serum, multiplex magnetic bead immunoassay kits were used as per manufacturer’s protocol (Invitrogen). Human cytokine 25-Plex panel was used to determine quantitative measurement for IL-10, IFN-γ, IL-2, IL-6, and TNF in study serum. sTNFRII and TGF-β were analyzed separately using singleplex bead kits. The serum samples were randomly assigned to the plates to avoid assay bias and to determine inter-assay differences. Five ascites samples which were quantified using cytometric bead array were also analyzed to determine limits of agreement. All samples were analyzed in duplicate and results were calculated as the average of two values. The samples were analyzed using a Luminex^®^ 200™ analyser (Luminex Corp.) as per standard protocol. Data were analyzed using a five-parametric-curve fitting within the manufacturer’s software.

Assessment of ascites and serum samples were on different platform as the samples were from part of a larger trial samples, where the respective platforms were used for cytokine assessment. The platforms were chosen based on the cytokines being analyzed. We formally compared the same samples run across both platforms to provide cross validation of the two using Bland–Altman method comparison study observed mean differences of estimated bias 2.04 ± 9.05 between two tests, and the 95% limits of agreement were between −15.7 and 19.8. Therefore, results from both the platforms were confirmed to be within good limits of agreement.

### Statistical Analysis

Comparison of two groups of data were analyzed by Wilcoxon matched-paired *t*-test, while three-group data were analyzed with one-way ANOVA with Dunn’s multiple comparison test (*post hoc*). Pearsons’ correlations were employed for correlation analyses. *p* < 0.05 was significant. Data were always shown as mean ± SEM. Statistical analyses were performed using Graphpad prism 7.0.

## Results

### Culturing PBMCs in Cell-Free Ovarian Cancer Malignant Ascites Increases TNFR2^+^ Expression on T Cell Subsets

**Figure 1:**
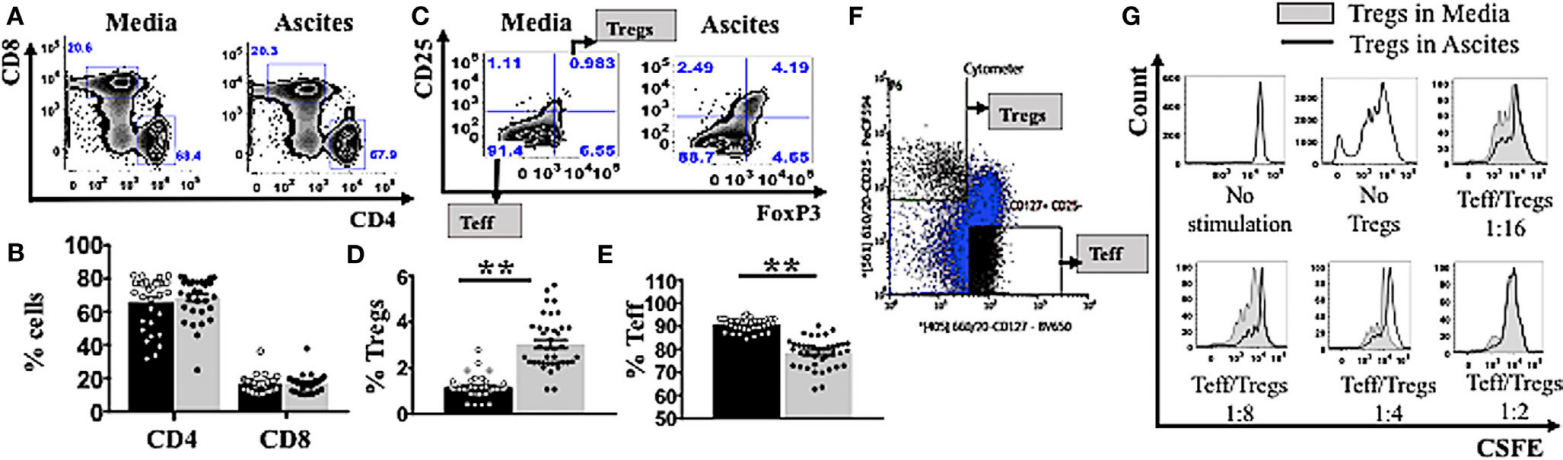
Ascites increases frequency of functional regulatory T cells (Tregs). **(A–E)** Peripheral blood mononuclear cells (PBMCs) from healthy donors (*n* = 30) were isolated by Ficoll density centrifugation and incubated *in vitro* in either AIM V media or cell-free ascites from advanced epithelial ovarian cancer patients for 48 h. Cells were washed, stained with anti-CD3, CD4, CD8, CD25, tumor necrosis factor receptor 2 (TNFR2), and FoxP3, and analyzed with flow cytometry. **(F,G)** PBMCs from two healthy donors were isolated by Ficoll density centrifugation. Cells were labelled anti-CD3, anti-CD4, anti-CD25, and anti-CD127, and flow cytometric cell sorting was performed. **(A)** Flow cytometry plots of CD4^+^ and CD8^+^ T cells within CD3^+^ T cells incubated in media and ascites. **(B)** The frequency (%) of CD4^+^ and CD8^+^ T cells within media (black bar) and ascites (gray bar) (*n* = 30). **(C)** Tregs were identified as CD25^hi^FoxP3^+^ and effector T cells (Teff) were identified as CD25^+^FoxP3^+^ within CD4^+^ T cells. Flow cytometry plots of Tregs and Teff cells within CD4^+^ T cells incubated in media and ascites. **(D,E)** The frequency (%) of Tregs **(D)** and Teff **(E)** within media (black bar) and ascites (gray bar) (*n* = 30). **(F)** Tregs and Teff were identified by gating on CD3^+^CD4^+^ population, followed by CD127 and CD25 to identify CD25^hi^CDl27^lo^ and CD25^−^CD127^+^ T cells respectively (*n* = 2). **(G)** Prior to suppression assay, Teff were labelled with carboxyfluorescein diacetate succinimidyl ester (CSFE) and incubated in media, while Tregs were cultured in either complete AIM V media or in cell-free ascites for 48 h (*n* = 2). Cells were then harvested and washed. The labelled effector cells were cultured either alone or at a 1:2, 1:4, 1:8, and 1:16 ratio with autologous pre-conditioned Tregs in a 96-well plate with anti-CD3/CD28 for 3 days. Cells were washed, stained, and analyzed by flow cytometry. Proliferation of Teff by Tregs incubated in ascites (black line) compared to Tregs incubated in media (gray line and shaded), cultured alone (no Tregs) or in co-culture with several ratios of Teff to Tregs. *p < 0.05 and **p < 0.0001, Wilcoxon matched-pair *t*-test. Data from PBMCs were pooled from three independent experiments (error bars-SEM), while data of sorted cells are replicates of two donors.

To quantify potential general changes in cell population frequencies in response to ovarian cancer ascites, we isolated peripheral mononuclear cells (PBMCs) from healthy donors (*n* = 30) and incubated them *in vitro* in AIM V media or cell-free ascites from advanced EOC patients, followed by cell staining and analysis by flow cytometry. T-lymphocytes were identified using an anti-CD3 antibody, a pan T-cell marker. The percentages of CD4^+^ and CD8^+^ T cells were unchanged following incubation of PBMCs in ascites (Figures 1A–B). Within the CD4^+^ gate, Tregs were further identified as CD25^hi^FoxP3^+^, while effector T cells (Teff) were identified as CD25^−^FoxP3^−^ (Figure 1C). CD25^hi^Foxp3^+^ cells gated using this strategy were further confirmed to be CD127^lo^ (Figure S1 in Supplementary Material). Malignant ascites compared to media induced higher frequencies of Tregs (Figure 1D) and conversely decreased frequencies of Teff (Figure 1E). As Treg-related cell markers can be expressed on non-Treg cells upon cell stimulation including transient expression of FoxP3 (76), we used purified Tregs to confirm that the phenotype of CD25^hi^FoxP3^+^ observed in ascites cultures identifies functional suppressors. We purified Tregs from PBMCs of two healthy donors (already conditioned in media or ascites for 48 h) by using flow cytometry cell sorting. Following gating on CD3^+^CD4^+^ lymphocyte population, Tregs were then sorted as CD25^hi^CD127^lo^ and Teff as CD25^−^CD127^+^ T cells (Figure 1F). The purity of post-sort Tregs were confirmed to be 95 ± 3% FoxP3^+^ by flow cytometry. We performed a suppression assay using these purified Tregs conditioned in ascites, to formally determine their ability to suppress autologous effector T cells compared to media. Purified CD4^+^CD25^−^ effector T cells were labeled with CSFE to monitor cell proliferation and were cultured alone (without Tregs) or at a 1:2, 1:4, or 1:8 and 1:16 ratio with autologous Tregs (pre-conditioned in either AIM V media or ascites for 48 h) in a 96-well plate with mAb stimulants anti-CD3 and soluble anti-CD28 for 3 days. As shown in Figure 1G, both media and ascites culture derived Tregs suppressed proliferation of Teffs. Moreover, on a cell-for-cell basis Tregs cultured in ascites showed higher suppressive capacity on autologous Teffs compared to Tregs conditioned in media (Figure 1G). The above result confirmed that malignant ascites caused increases in functional Tregs.

**Figure 2:**
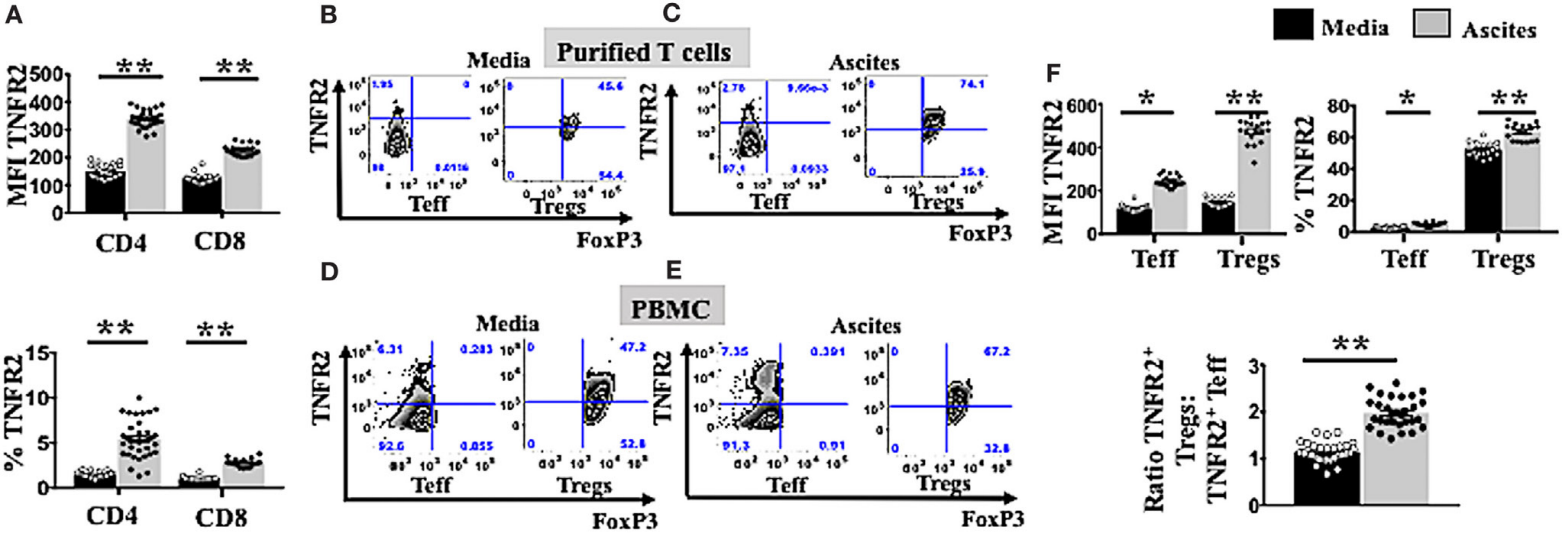
Ascites upregulates tumor necrosis factor receptor 2 (TNFR2) expression on both T effector (Teff) and regulatory T cells (Tregs). **(A)** Peripheral blood mononuclear cells (PBMCs) from healthy donors (*n* = 30) were isolated by Ficoll density centrifugation and incubated *in vitro* in either AIM V media or cell-free ascites from advanced epithelial ovarian cancer (EOC) patients for 48 h. Cells were washed, stained with anti-CD3, CD4, and CD8 and TNFR2 and analyzed with flow cytomcery **(A)**. The level of TNFR2 expression (median fluorescence intensity, MFI) and frequency (%) of TNFR2 within CD4^+^ and CD8^+^ T cells in media (black bar) and ascites (gray bar) (*n* = 30) **(B,C)**. PBMCs from two healthy donors were isolated by Ficoll density centrifugation. Cells were labelled anti-CD3, anti-CD4, anti-CD25, and anti-CD127 and flow cytometric cell sorting for Tregs and Teff was performed. Isolated Tregs and Teff were cultured in either complete AIM V media or in cell-free ascites for 48 h. Cells were then washed and labeled for surface markers with CD3, CD4, CD25, and TNFR2 followed by intracellular staining for FoxP3 and prepared for flow cytometric analysis **(B,C)**. Representative flow cytometry expression of TNFR2 and FoxP3 on sorted Tregs and Teff cells as well incubated in media **(B)** and ascites **(C)**. **(D–F)** PBMCs from a single healthy donor were incubated *in vitro* for 48 h in either AIM V media or cell-free ascites from 18 patients with advanced EOC (*n* = 18). Cells were washed, stained with CD3, CD4, CD25, TNFR2, and FoxP3, and analyzed with flow cytometry. **(D,E)** Expression of TNFR2 and FoxP3 was analyzed with flow cytometry by gating on Tregs and Teff cells derived from PBMCs incubated in media and ascites. **(F)** The level of TNFR2 expression (MFI) within Teff and Tregs and percentages (%) of TNFR2^+^ Teff and Tregs and the ratio of TNFR2^+^ Tregs to TNFR2^+^ Teff in media and ascites (*n* = 18). *p < 0.05 and **p < 0.0001, Wilcoxon matched-pair *t*-test. Data from PBMCs were pooled from three independent experiments (error bars-SEM), while data of sorted cells are replicates of two donors.

We next investigated whether culturing in ascites also influenced changes in the phenotypes of Tregs and Teffs. We incubated PBMCs from healthy donors (*n* = 30) in AIM V media or cell-free malignant ascites, followed by cell staining and analysis by flow cytometry. We found that culture with ascites strongly upregulated TNFR2 expression on both CD4^+^ and CD8^+^ T cell subsets (Figure 2A), resulting in a higher proportion (%) of TNFR2^+^ CD4 and CD8 T cells (Figure 2A). Additionally, stimulation with malignant ascites significantly upregulated the median fluorescence intensity of TNFR2 on Tregs as well as the frequency of TNFR2^+^ Tregs (Figure 2F) (*p* < 0.05). This effect was also found consistently in 18 separate EOC patient derived ascites affecting PBMCs from a single healthy volunteer (Figure 2F) or a single cell-free ascites affecting PBMCs from 30 healthy volunteers (data not shown). We also used purified Tregs and Teff from healthy donors (*n* = 2) and cultured them in a similar *in vitro* system. We observed similar increased in TNFR2 expression on these purified T cell subsets, particularly Tregs (Figures 2B,C). Although ascites decreased the frequency of Teff (Figure 1E), ascites significantly increased the frequency of TNFR2^+^ Teffs, as well as the level of expression of TNFR2 on the effectors (Figures 2D–F). However, the fold change in TNFR2 expression in ascites compared to media was significantly lower for Teffs compared to Tregs (1.97 ± 0.37 vs 3.37 ± 0.48, *p* < 0.001) resulting in a significantly increased ratio of TNFR2^+^ Treg/Teff (1.14 ± 0.21 vs 1.98 ± 0.32, *p* < 0.0001) (Figure 2F). Together the above data demonstrate conditioning in ascites promotes higher expression of TNFR2 on Tregs and Teffs.

### TNFR2^+^ Tregs Conditioned in Malignant Ascites Express Higher Levels of Immunosuppressive Molecules PD-L1, CTLA-4, and GARP and Are Negatively Correlated with Total and TNFR2^+^ Teff Activity

**Figure 3:**
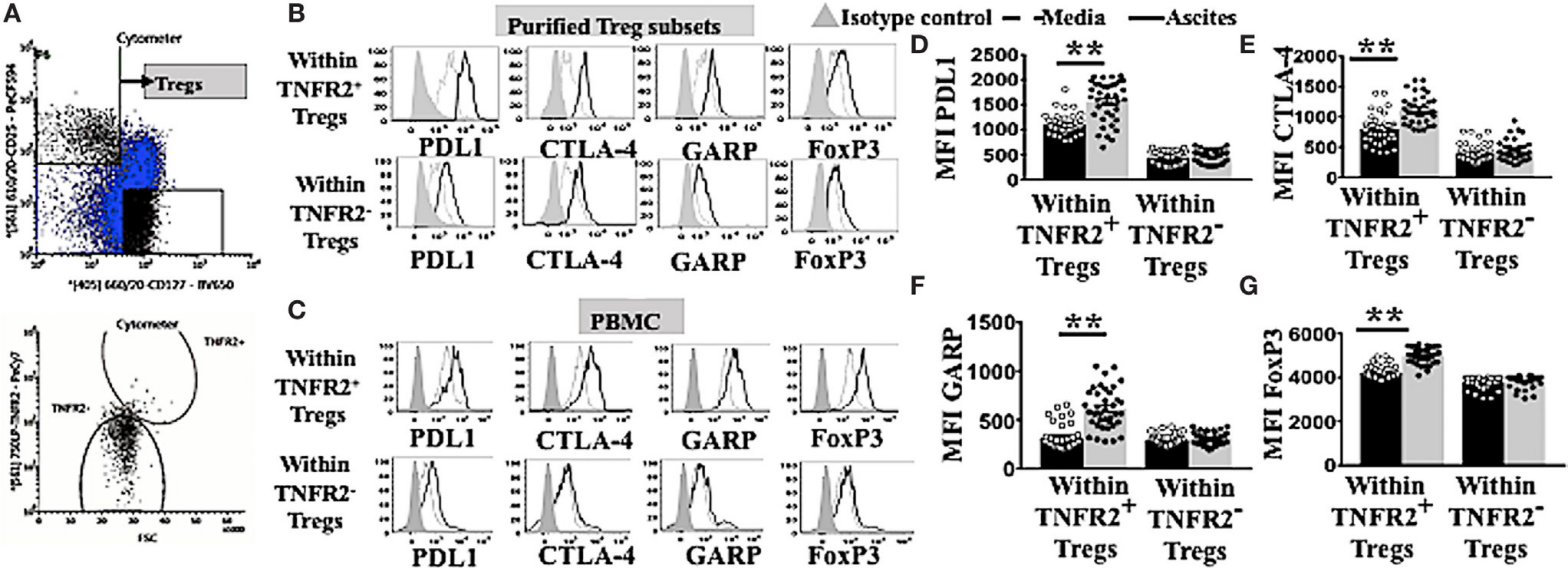
TNFR2^+^ regulatory T cells (Tregs) conditioned in ascites expressed higher levels of functional immunosuppressive molecules, such as programmed cell death ligand-1 (PD-L1), CTLA-4, and GARP. **(A)** Peripheral blood mononuclear cells (PBMCs) from two healthy donors were isolated by Ficoll density centrifugation and labelled with anti-CD3, CD4, CD25. CD127 and tumor necrosis factor receptor 2 (TNFR2) and flow cytometric cell sorting was performed. **(A)** Tregs were identified by gating on CD3* CD4* population, followed by CD127 and CD25 to identity CD25^hi^ CD127^lo^. Two populations of Tregs were sorted by gating on TNFR2^+^ and TNFR2^−^. Isolated Tregs subsets were cultured in either complete AIM V media or in cell-free ascites for 48 h. Cells were washed and labeled with anti-CD3, CD4. CD25, PD-L1, CTLA-4. GARP and FoxP3 and prepared for flow cytometric analysis. **(C–G)** PBMCs from healthy donors (*n* = 30) were incubated in either AIM V media or ascites supernatant from an advanced epithelial ovarian cancer patient for 48 h. Cells were washed, stained for anti-CD3, CD4, CD25, TNFR2. PD-L1, CTLA-4, GARP, and FoxP3, and prepared for flow cytometric analysis. **(C,B)** Expression of PD-LI, CTLA-4, GARP, and FoxP3 expression were analyzed in sorted TNFR2^+^ Tregs and TNFR2^−^ Tregs, **(B)** and in Tregs from PBMCs by gating on Tregs TNFR2^+^ and Tregs TNFR2^−^ cells incubated in media (dashed line) and ascites (solid line). The shaded histogram represents staining with an isotype control. **(D–G)** The level of expression (in MFI) PD-L1, CTLA-4, GARP, and FoxP3 within Tregs TNFR2^+^ and Tregs TNFR2^−^ cells incubated in media (black bar) and ascites (gray bar) (*n* = 30). *p < 0.05 and **p < 0.0001, Wilcoxon matched-pair *t*-test. Data using PBMCs were pooled from three independent experiments (error bars-SEM), while data of sorted cells are replicates of two donors.

TNFR2^+^ Tregs have been demonstrated to be more suppressive compared to TNFR2^−^ Tregs in various diseases including chronic inflammatory conditions (16–19). Highly suppressive Tregs usually express elevated levels of functional immunosuppressive molecules CTLA-4, GARP (13, 18, 19), and PD-L1 (34). We explored whether ascites could further induce other immunosuppressive molecules on TNFR2^+^ and TNFR2^−^ Tregs. We used purified Tregs TNFR2^+^ and Tregs TNFR2^−^ subsets isolated from PBMC of healthy donors (*n* = 2) by flow cytometric cell sorting. These purified Treg subsets were initially identified by gating on CD3^+^CD4^+^ for lymphocytes population and followed by CD25^hi^CD127^lo^ (Figure 3A). Two subsets of Tregs were then derived by sorting on TNFR2^+^ and TNFR2^−^ population. Following incubation in either AIM V media or cell-free ascites of an advanced EOC patient for 48 h, purified Tregs were stained for surface markers with CD3, CD4, CD25, PD-L1, CTLA-4, GARP followed by intracellular staining for FoxP3 and prepared for flow cytometric analysis. This exploratory data using purified Treg subsets suggested that TNFR2^+^ Tregs conditioned in ascites had increased expression of immunosuppressive molecules, including PD-L1, CTLA-4, and GARP when compared to TNFR2^+^ Tregs in media as well as when compared to TNFR2^−^ Tregs (Figure 3B).

We, therefore, performed an experiment to determine if this upregulation pattern would also be reproducibly found when using PBMC. We incubated PBMCs from healthy donors (*n* = 30) in either AIM V media or ascites supernatant of an advanced EOC patient for 48 h followed by cell labeling with anti-CD3, CD4, CD25, TNFR2, PD-L1, CTLA-4, GARP, and FoxP3 and subsequent flow cytometric analysis. TNFR2^+^ Tregs conditioned in malignant ascites showed significantly higher levels of expression of PD-L1 (1,550 ± 66.0 vs 1,103 ± 34. 2, *p* < 0.0001), CTLA-4 (1,116 ± 36.6 vs 795 ± 39.5, *p* < 0.0001), and GARP (612.5 ± 34.0 vs 315.7 ± 20.9, *p* < 0.0001) compared to media (Figure 3C). Moreover, within PBMCs conditioned in ascites, TNFR2^+^ Tregs expressed higher levels of immunosuppressive molecules compared to TNFR2^−^ Tregs, including PD-L1 (1,898 ± 59.9 vs 689 ± 29.5, *p* < 0.0001), CTLA-4 (1,082 ± 32.6 vs 465.5 ± 37.8, *p* < 0.0001), and GARP (544.3 ± 35.3 vs 308.8 ± 10.2, *p* < 0.0001) (Figures 3C–F). These differences were likely to be driven by significantly higher expression of FoxP3 on TNFR2^+^ Tregs conditioned in ascites than media (5,048 ± 48.4 vs 4,238 ± 56.5, *p* < 0.0001), and in TNFR2^+^ compared to TNFR2^−^ Tregs within ascites conditioned cells (Figures 3C,G) (5,048 ± 48.4 vs 3,842 ± 32.0, *p* < 0.0001). The above data support a more potent immunosuppressive regulatory phenotype for TNFR2^+^ Tregs conditioned in ascites as compared to media, as well as for TNFR2^+^ compared to TNFR2^−^ Treg induced by culturing PBMCs in ascites.

**Figure 4:**
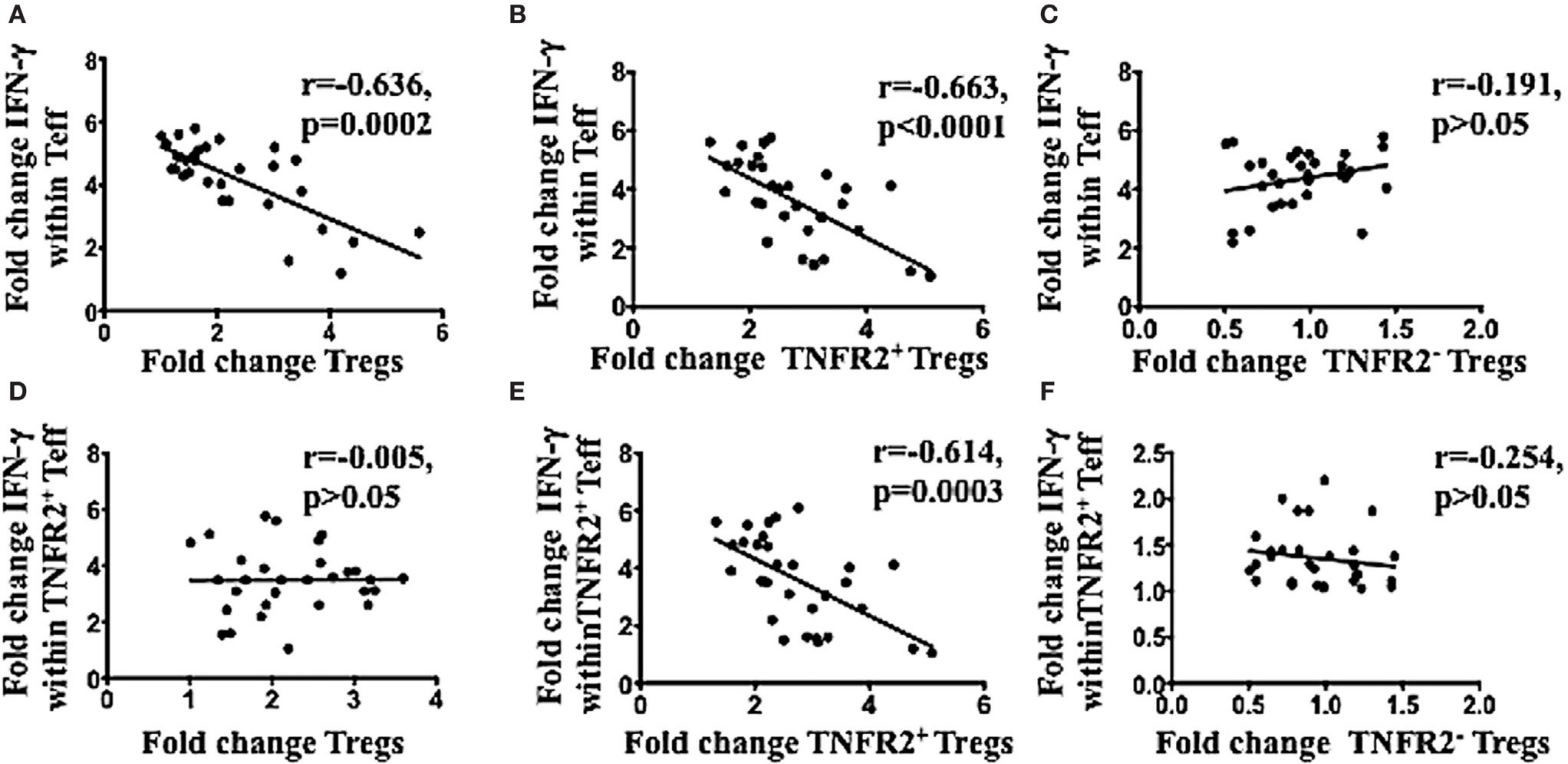
TNFR2^+^ regulatory T cells (Tregs) conditioned in ascites, compared to media had a higher frequency and were inversely correlated with IFN-γ production by effector T cells in the same donor. Peripheral blood mononuclear cells from healthy donors from three independent experiments (*n* = 30) were incubated in either AIM V media or ascites supernatant of an advanced epithelial ovarian cancer patient for 48 h in two plates simultaneously. One of plate was left unstimulated for quantification of T cell subsets, while another plate was incubated with PMA and ionomycin for the last 6 h, and Brefeldin A was added at 1 μg/ml for the last 5 h of incubation, to detect intracellular cytokine production. Cells were washed, labeled for surface markers CD3, CD4, CD25. Tumor necrosis factor receptor 2 (TNFR2) followed by intracellular staining for FoxP3 and IFN-gamma (IFN-γ) and then prepared for flow cytometric analysis. **(A–C)** Correlation of the fold change in Tregs **(A)**. TNFR2^+^ Tregs **(B)**, and TNFR2^–^ Tregs **(C)**, following conditioning in ascites with the fold change in IFN-γ production by T effector (Teff) respectively. **(D–F)** Correlation of the fold change in Tregs **(D)**, TNFR2^+^ Tregs **(E)**, and TNFR2^–^ Tregs **(F)** conditioned in ascites with the fold change in IFN-γ production by TNFR2^+^ Teff respectively. *p* > 0.05 is not significant.

The latter hypothesis was specifically tested by determining the capacity of the ascites induced TNFR2^+^ Tregs to be associated with decreased CD4 effector T cell function. PBMCs from healthy donors (*n* = 30) were incubated in either AIM V media or cell-free malignant ascites in two plates in parallel. One plate remained unstimulated to assess the proportion of T cell subsets, while another plate underwent PMA and ionomycin stimulation to detect intracellular cytokine production. As expected, Tregs were induced at a higher frequency by ascites compared to media in unstimulated PBMCs cultures with ascites, and this increase in Tregs (fold change) (media or ascites cultured) was inversely correlated with the ability of Teff to produce IFN-γ (Figure 4A). Moreover, only increases TNFR2^+^ Tregs (Figure 4B), but not TNFR2^−^ Tregs (Figure 4C) were inversely correlated with effector CD4 T cell function. Given we found ascites also increased TNFR2^+^ Teffs, and these cells are reported as hyperactive cytokine producers (77), we tested whether conventional Tregs would be able to suppress their cytokine production. Figure 4D shows that there was no inverse relationship between conventional Tregs increases and TNFR2^+^ Teff IFN-γ production, showing they could not suppress this effector T cell subset. Previous studies by Chen and colleagues have suggested only TNFR2^+^ Tregs can suppress TNFR2^+^ T cell effectors (78). Consistent with these studies the fold change of TNFR2^+^ Tregs (Figure 4E), but not TNFR2^−^ Tregs (Figure 4F) was strongly inversely correlated with the ability of TNFR2^+^ Teffs to produce IFN-γ. We next determined whether the proportion of Tregs, TNFR2^+^ Tregs, and TNFR2^−^ Tregs correlated with the total production of IFN-γ within CD4^+^ T cells to ensure that the inverse correlation seen is not influenced by reciprocal gating for Teff and Tregs subpopulations following incubation in ascites. There was inverse correlation between total IFN-γ within CD4^+^ T cells to Tregs and TNFR2^+^ Tregs, but not TNFR2^−^ Tregs. There was also increase in the fold change of IFN-γ producing CD4^+^TNFR2^+^ T cells which inversely correlated to Tregs as well as TNFR2^+^ Tregs but not TNFR2^−^ Tregs (Figure S2 in Supplementary Material). Similar correlation patterns were also observed when the level of IFN-γ expression measured by mean fluorescence intensity (MFI) was used instead of proportion of IFN-y^+^ T cells (data not shown). We also looked at whether CD8 T cell function was affected by the induced TNFR2^+^ Tregs. There was no inverse correlation between conventional Tregs or TNFR2^+^ Tregs with the ability of CD8 to produce IFN-γ (data not shown).

### Higher Levels of Immunosuppressive (sTNFR2, IL-10, and TGF-β) and Pro-inflammatory Cytokines (IL-6 and TNF) Are Present in Malignant Ascites Compared to Serum of Advanced Ovarian Cancer Patients

**Figure 5:**
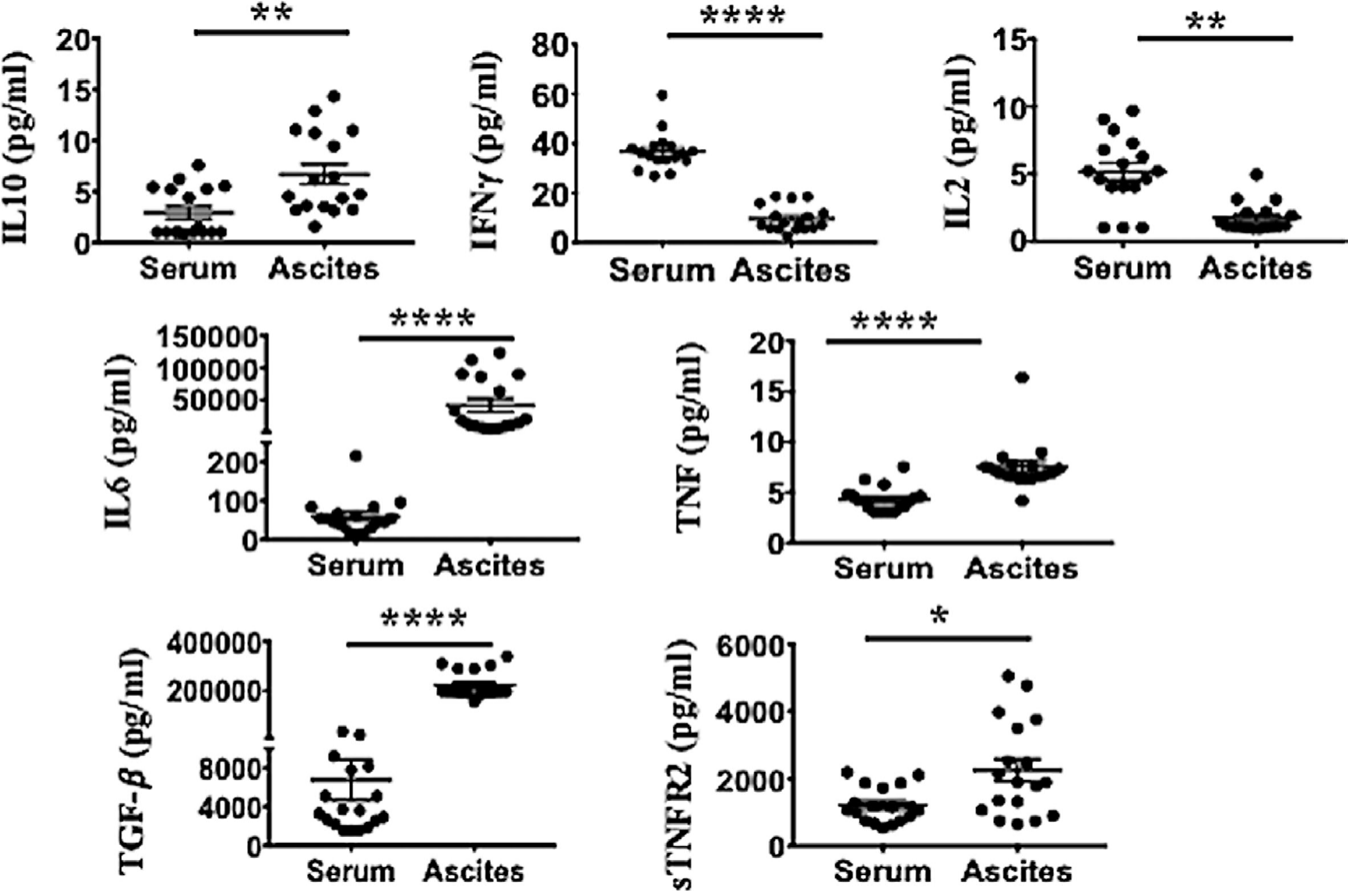
Ascites of epithelial ovarian cancer (EOC) patients contain higher levels of immunosuppressive (sTNFR2, IL-10 and TGF-β) and pro-inflammatory cytokines [interleukin 6 (IL-6) and TNF], but lower levels of IFN-γ compared to their respective serum. Cell-free ascites and corresponding peripheral blood serum were collected from 18 patients with advanced EOC and soluble biomarkers including IL-10, IFN-γ, IL-2, IL-6, TNF, TGF-β, and sTNFR2 quantified using multiplexed bead-based immunoassay. *p < 0.05, **p = 0.001–0.01, ***p = 0.0001–0.001, ***p < 0.0001, Wilcoxon matched-paired *t*-test (error bars-SEM).

We next determined the pro-inflammatory soluble factors present in malignant ascites that may promote TNFR2^+^ Tregs. Ascites and respective peripheral blood serum were collected from 18 patients with ovarian cancer. The mean age at the time of cytoreductive surgery was 60.2 ± 7.82 years (range 49–78 years). All patients had advanced disease including 16 patients with Stage III (88.8%) and 2 patients (11.2%) with Stage IV disease. Specimens were collected prior to surgery and before chemotherapy. Final pathology was consistent with high-grade (poorly differentiated) serous type ovarian cancer. We performed multiplexed bead-based immunoassay on cell-free pre-treatment ascites and on respective sera of all 18 patients. Ascites specimen demonstrated a significant elevated level of immunosuppressive (sTNFR2, IL-10 and TGF-β) and pro-inflammatory cytokines (IL-6 and TNF) compared to respective serum (Figure 5). TNFR2 expression has been shown to be induced by either TNF (15, 79) or TNF in combination with IL-6 in cancer cell lines (20). We next explored whether blockade of either of these soluble factors would influence overall Treg induction by malignant ascites, as well as TNFR2 expression.

### TNFR2^+^ Tregs Are Decreased in Frequency and in their Suppressive Phenotype following Blockade of IL-6 in Ascites

**Figure 6:**
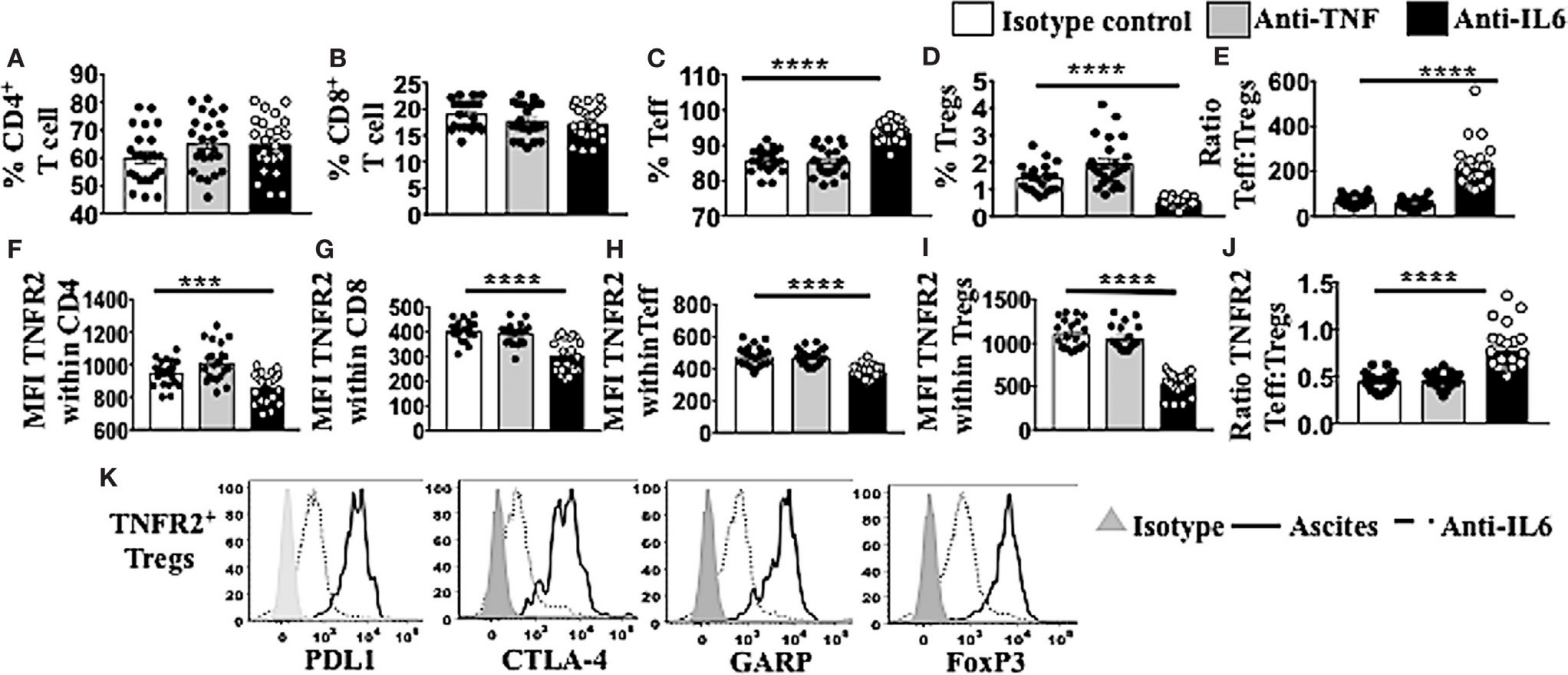
Blockade of bioctive interleukin 6 (IL-6), but not TNF, within ascites of epithelial ovarian cancer (EOC) patients decreases percentage of regulatory T cells (Tregs) as well as TNFR2^+^ Tregs, as well as tumor necrosis factor receptor 2 (TNFR2) expression. Peripheral blood mononuclear cells (PBMCs) from healthy donors (*n* = 30) were isolated by Ficoll density centrifugation and incubated *in vitro* in either AIM V media or cell-free ascites from advanced EOC patients for 48 h. IL-6 and tumor necrosis factor (TNF) within the ascites were blocked with mouse anti-human IL-6 monoclonal antibody (mAb) at 2.5 μg/ml and mouse anti-human TNF at 500 ng/ml. Mouse IgG1 immunoglobulins (isotype control) were used as a negative control. Cells were washed, labeled for surface and intracellular markers CD3, CD4, CD8, CD25, TNFR2, programmed cell death ligand-1 (PD-L1), CTLA-4, GARP, and FoxP3, and analyzed with flow cytometry. **(A–E)** The frequency (%) of CD4, CD8, T effector (Teff), and Treg **(A–D)** and the ratio of Teff to Tregs **(E)** within ascites following blockade with IL-6 (black bar) or TNF (gray bar). **(F–J)** The level of TNFR2 expression (median fluorescence intensity, MFI) within CD4, CD8, Teff, and Tregs **(F–I)** and ratio of TNFR2 expressing Teff to Tregs **(J)** in ascites following IL-6 or TNF blockade. **(K)** PD-L1, CTLA-4, GARP, and FoxP3 expression on TNFR2^+^ Tregs cells incubated in ascites (solid line) and following blockade with IL-6 mAb (dashed line). The shaded (gray) histogram represents staining with an isotype control. *p < 0.05, **p = 0.001–0.01, ***p = 0.0001–0.001, ****p < 0.0001, One-way ANOVA and Dunn’s multiple comparison test (post hoc). Data are pooled from three independent experiments (error bars-SEM).

Following conditioning of PBMCs from healthy donors in malignant cell-free ascites for 48 h, soluble factors IL-6 and TNF within the ascites were blocked with neutralizing mAb against IL-6 or TNF. Mouse IgG1 immunoglobulins (isotype control) (R&D USA) were used as a negative control. Cells were washed, stained, and analyzed using flow cytometry. The overall fraction of CD4^+^ and CD8^+^ T cells following conditioning in ascites and blockade with either IL-6 or TNF mAbs showed no significant differences compared to isotype control (Figures 6A,B). By contrast, within CD4 T cells, there was a clear increase in Teff and decrease in Tregs following blockade of IL-6 within ascites compared to isotype control (Figures 6C,D). The ratio of Teff to Tregs was, therefore, greatly increased following blockade of IL-6 within ascites (Figure 6E). These effects were not observed following blockade of TNF within ascites. Moreover, blockade of bioactive IL-6 in ascites, but not TNF, also decreased the level of TNFR2 expression in both CD4^+^ and CD8^+^ T cells (Figures 6F,G) as well as Teff and Tregs (Figures 6H,I), with the most prominent effect found on Tregs, overall increasing the ratio of TNFR2^+^ Teffs to TNFR2^+^ Tregs (Figure 6J). Following the blockade of bioactive IL-6 within ascites with monoclonal antibody, the level of expression of PD-L1 (1,550 ± 66.0 vs 803 ± 104.8, *p* < 0.0001), CTLA-4 (1,116 ± 36.6 vs 203.2 ± 42.0, *p* < 0.0001), and GARP (612.5 ± 34.0 vs 58.0 ± 32.8, *p* < 0.0001) decreased within TNFR2^+^ Tregs conditioned in ascites compared to media (Figure 6K). The expression of FoxP3 on TNFR2^+^ Tregs following blockade of IL-6 was decreased compared to TNFR2^+^ Tregs within ascites conditioned cells (Figure 6K). The above data support an active role of IL-6 within ascites on promoting TNFR2 expression on Tregs.

## Discussion

The present study shows for the first time that culture with ovarian cancer-associated malignant ascites promotes increased frequencies of T cells expressing high levels of TNFR2. Moreover, it shows a critical role for IL-6 in promoting TNFR2 expression on T cells, and particularly on Tregs. These findings could provide a mechanistic link between IL-6 and Tregs, where both have been independently associated with disease progression, for example in breast, lung, renal, and colorectal cancer (80–83). These results also suggest that IL-6 blocking therapy may have additional beneficial therapeutic effects (62). Specifically, and similarly to our *in vitro* studies, it may preferentially decrease TNFR2^+^ expressing Tregs, leading to an increase in IFN-γ production by Teffs. This is particularly important in the case of TNFR2^+^ Teffs, since our studies show that, similarly to what has been demonstrated in autoimmunity (84), that these cells may only be suppressed by TNFR2^+^ Tregs within ovarian cancer ascites.

The high levels of TNFR2^+^ Tregs within patient ascites have been suggested to be the consequence of preferential migration of TNFR2^+^CCR4^+^ Tregs into ascites (9, 18). Herein, we show for the first time that a higher fraction of TNFR2^+^ expressing T cell subsets, particularly on CD4^+^CD25^hi^FoxP3^+^ (Tregs) can also be induced *de novo* from healthy donor PBMCs conditioned with cell-free ovarian cancer ascites. In addition, the increase in TNFR2 expression also demonstrated on purified Treg subsets cultured in ascites, further suggests the direct induction of TNFR2 by soluble factors in the ascites in the absence of other immune cells. The ovarian cancer ascites used in our study from patients in advanced disease stages showed a mixture and levels of soluble factors similar to previous studies (11, 18, 85, 86), with a predominance toward Th2 vs Th1 type cytokines, and high levels of IL-6 and TNF compared to serum. We have consistently observed similar effects across 18 different ovarian cancer ascites and on cells derived from different human volunteers, confirming the robustness of this effect.

In addition to being induced at high frequencies by ovarian cancer ascites, TNFR2^+^ Tregs were also potent suppressors when compared to the TNFR2^−^ T cell fraction, which was in agreement with previous studies (13, 18). The higher frequency of TNFR2^+^ Tregs induced from malignant ascites was inversely correlated with IFN-γ production by effector T cells in our study. TNFR2^+^ Tregs conditioned in ovarian cancer ascites, compared to media, also expressed higher levels of functional immunosuppressive molecules such as PD-L1, CTLA-4, and GARP. These differences were likely to be driven by the significantly higher expression of FoxP3 on TNFR2^+^ Tregs compared to TNFR2^−^ Tregs within ascites conditioned cells. Our observation that only TNFR2^+^ Tregs suppress TNFR2^+^ Teff in these cultures is highly likely to be mediated by the higher levels of these immunosuppressive molecules. Future studies may be able to distinguish the relative contribution of each one of these molecules on their ability to suppress TNFR2^+^ effectors. The present study shows that the high levels of CTLA-4 and GARP, previously shown to be present on Tregs derived from human cancer ascites (18), may be directly induced by soluble factors within this ascites, and specifically IL-6. Moreover, PD-L1 expression is also upregulated by ovarian cancer ascites. Recent human trials have demonstrated promising antitumor efficacy for immunosuppressive checkpoint inhibitors in several cancer types (35–37); however, their role as ovarian cancer therapeutics is still not well-established. As blockade of bioactive IL-6 within ascites decreased TNFR2 expression on Tregs, IL-6 may potentially play a combined role with other pro-inflammatory cytokines such as TNF, as seen in past studies (38), to help decrease immunosuppressive molecules such as CTLA-4, GARP, and PD-L1.

Tumor necrosis factor receptor 2 expression can also be induced on Teff by soluble factors within the tumor microenvironment or by TCR stimulation according to Chen (78), and this was also observed in our study. The expression of TNFR2 on Teffs rendered these cells resistant to suppression by Tregs, whereas TNFR2-expressing Teff were highly proliferative, secreted high levels of effector cytokines (IFN-γ), and were more resistant to Treg-mediated inhibition (78). This was also demonstrated in our study where TNFR2^+^ effector T cells were resistant to suppression by TNFR2^−^ Tregs, but susceptible to inhibition by TNFR2^+^ Tregs.

Tumor necrosis factor and IL-6 are pleiotropic pro-inflammatory cytokines that modulate growth, differentiation, and proliferation of various types of cells. Both these cytokines, secreted by cancer cells as well as immune cells, are also involved in the inflammation process and play a functional role in malignancy promotion and progression in various cancers including ovarian cancer (61, 63, 87–89). The present study shows a new critical role for IL-6 present in ascites in promoting TNFR2 expression on T cells, particularly Tregs. In contrast to previous studies, TNF did not appear to be similarly critical. The role of TNF and IL-6, independently or in combination, on TNFR2 expression on diverse cell types is controversial (20, 64, 79, 90). Addition of exogenous TNF, but not IL-6 or IL-1β, have been found to upregulate TNFR2 on murine Treg cells *in vitro* (64). TNF alone has no effect on TNFR2 expression on human colon cancer cell lines *in vitro*; however, the combination of IL-6 and TNF can upregulate TNFR2 on such cells (90). In the same study, the regulation of TNFR2 by IL-6 was confirmed using an *in vivo* murine model. The lack of IL-6 in TCR/IL-6 KO double mutant (IL-6 DKO) mice resulted in markedly reduced TNFR2 expression in colonic epithelial cells compared with TCR KO mice, even in the presence of mild colitis in both groups (90). It was, therefore, possible that, even if we could not detect direct effects for TNF, TNF would still potentiate TNFR2 induction by IL-6. However, we did not observe an enhancement of the decrease in TNFR2 expression by blockade of both IL-6 and TNF compared to IL-6 blockade alone (data not shown).

Tumor necrosis factor receptor 2 upregulation is associated with increased suppressive activity of Tregs. Chen and colleagues used purified T cell subsets from lymph nodes of C57/BL6 mice and performed a suppression assay by coculturing Tregs and Teff with exogenous TNF (10 ng/ml) and IL-2 (10 ng/ml) for up to 72 h. The authors found that prolonged exposure to TNF increased Treg suppressive activity, but shorter duration did not demonstrate this effect (64). We did not find a prominent role for TNF in our study, most likely given we used different methods (ascites vs pure cytokines), species (murine vs human), measurements (TNFR2 expression vs suppression add-back assays), and timepoints (48 vs 72 h). Ascites may contain other soluble factors such as TGF-β that may synergize with IL-6 to promote TNFR2 expression. TGF-β has been suggested to play a role in the generation and expansion and stability of Tregs (91). The exploration of the influence of these cytokines in promoting TNFR2 expression is worthwhile, but beyond the scope of this paper. Preliminary results suggest TGF-β may not play a substantial role in the upregulation of TNFR2 on Tregs (manuscript in preparation). In the present paper, we found that blockade of the elevated IL-6 level within ascites, but not TNF, decreased TNFR2 expression, particularly on Tregs.

Therefore, the findings from this study suggest IL-6 may play an active role in promoting early TNFR2 upregulation, which may in turn enable these Tregs to respond to TNF. The nature of our short term cultures (we do not add IL-2 or other factors which may extend cell survival) does not allow us to test this hypothesis directly. However, taking together with the findings from a previous study (64) and the present data, we propose IL-6 may promote early activation of TNFR2 expression on Tregs, while TNF may be responsible for long term maintenance of TNFR2 expression and Tregs suppressive activity. Consistent with this hypothesis, Mizoguchi and colleagues have observed in their *in vitro* system using colonic epithelial cells from murine colitis model that while TNFR2 upregulation is seen on day 8, upregulation of IL-6/STAT3 is observed earlier by day 4, indicating that the IL-6/STAT3 signaling cascade is activated before upregulation of TNFR2 expression (90). Another recent study looking at human colon cancer cell lines (20), confirmed the critical role for the IL-6/STAT3 pathway in enabling TNFR2 upregulation (20, 90).

Future studies could also address the influence of IL-6 on different subsets of TNFR2 expressing effector T cells. Previous studies have shown that TNFR2^+^ effectors were capable upon anti-CD3 and anti-CD28 stimulation of producing a range of cytokines including IFN-y, IL-2 and IL-10 (77, 78). It would be particularly interesting to study the effect of IL-6 on cytokine producing Th17 cells given literature suggesting that Th17 plays an important role in the pathogenesis of diverse group of autoimmune diseases as well inflammatory diseases and cancers, including ovarian cancer (92, 93).

The immune system is capable of effective antitumor responses against many cancers including ovarian cancer. As ovarian cancer has been identified as an immunogenic tumor, immunotherapy should be optimized to be included as part of ovarian cancer therapeutics. Our study demonstrates that in ovarian cancer, TNFR2 expression can be selectively decreased on all T cells, and predominantly on Tregs, by blockade of bioactive IL-6 within ascites. Our findings support future research into two potential interactive immunotherapeutic targets, TNFR2^+^ Tregs and IL-6, to help enhance effective antitumor responses in patients with ovarian cancer.

## Ethics Statement

This study was carried out in accordance with the recommendations of Immunity and Ovarian Cancer trial (Project 13/32), HREC of Royal Women’s Hospital with written informed consent from all patients. All patients gave written informed consent in accordance with the Declaration of Helsinki. The protocol was approved by HREC of the Royal Women’s Hospital, Melbourne.

## Author Contributions

Concept and design: NK, MM, OM, MQ, and MP. Development of methodology: NK, MM, MQ, and MP. Acquisition of data (acquired and managed patients, provided facilities, etc.) and writing, review, and/or revision of the manuscript: NK, MM, OM, AS, MQ, and MP. Analysis and interpretation of data (e.g., statistical analysis, computational analysis): NK, MM, and MP. Administrative, technical, or material support (i.e., organizing data, constructing databases): NK, OM, AS, MQ, and MP. Study supervision: OM, MQ, and MP.

## Conflict of Interest Statement

The authors declare that the research was conducted in the absence of any commercial or financial relationships that could be construed as a potential conflict of interest.
