# Supplementary figures and images for: Interleukin 6 Present in Inflammatory Ascites from Advanced Epithelial Ovarian Cancer Patients Promotes Tumor Necrosis Factor Receptor 2-Expressing Regulatory T Cells
Source: Front Immunol. 2017 Nov 6;8:1482. doi: 10.3389/fimmu.2017.01482 (PMC5681739; doi:10.3389/fimmu.2017.01482)

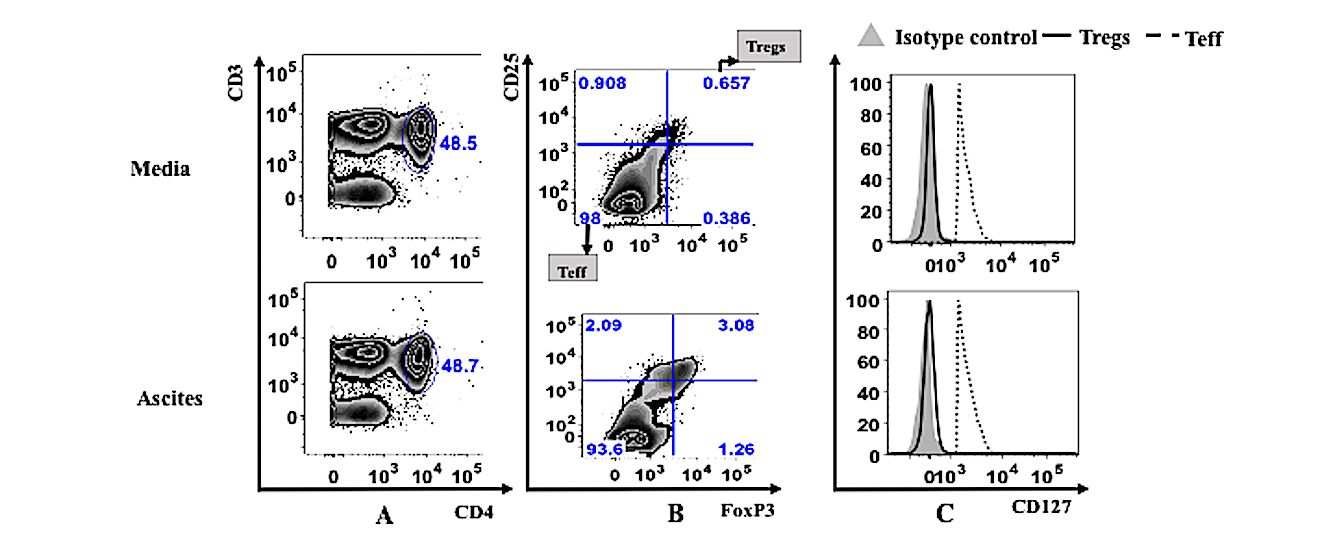

Supplement: Supplementary file 1 [file Image_1.tiff]

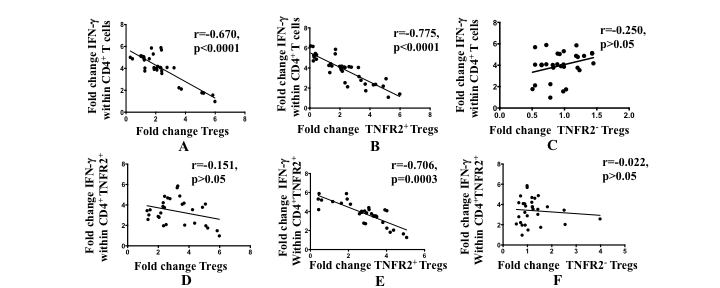

Supplement: Supplementary file 2 [file Image_2.tiff]
